# Key feature-cases as virtual patients in education of veterinary neurology

**DOI:** 10.3389/fvets.2022.911026

**Published:** 2022-08-19

**Authors:** Solveig Brigitta Reeh, Christin Kleinsorgen, Elisabeth Schaper, Holger Andreas Volk, Andrea Tipold

**Affiliations:** ^1^Department of Small Animal Medicine and Surgery, University of Veterinary Medicine, Hannover, Germany; ^2^Center for E-Learning, Didactics and Educational Research, University of Veterinary Medicine, Hannover, Germany

**Keywords:** Key feature cases, virtual patients, Clinical Reasoning, veterinary neurology, clinical decision-making

## Abstract

To provide students of veterinary medicine with the necessary day 1 competences, e-learning offerings are increasingly used in addition to classical teaching formats such as lectures. For example, virtual patients offer the possibility of case-based, computer-assisted learning. A concept to teach and test clinical decision-making is the key feature (KF) approach. KF questions consist of three to five critical points that are crucial for the case resolution. In the current study usage, learning success, usability and acceptance of KF cases as neurological virtual patients should be determined in comparison to the long cases format. Elective courses were offered in winter term 2019/20 and summer term 2020 and a total of 38 virtual patients with neurological diseases were presented in the KF format. Eight cases were provided with a new clinical decision-making application (Clinical Reasoning Tool) and contrasted with eight other cases without the tool. In addition to the evaluation of the learning analytics (e.g., processing times, success rates), an evaluation took place after course completion. After 229 course participations (168 individual students and additional 61 with repeated participation), 199 evaluation sheets were completed. The average processing time of a long case was 53 min, while that of a KF case 17 min. 78% of the long cases and 73% of KF cases were successfully completed. The average processing time of cases with Clinical Reasoning Tool was 19 min. The success rate was 58.3 vs. 60.3% for cases without the tool. In the survey, the long cases received a ranking (1 = very good, 6 = poor) of 2.4, while KF cases received a grade of 1.6, 134 of the respondents confirmed that the casework made them feel better prepared to secure a diagnosis in a real patient. Flexibility in learning (*n* = 93) and practical relevance (*n* = 65) were the most frequently listed positive aspects. Since KF cases are short and highlight only the most important features of a patient, 30% (*n* = 70) of respondents expressed the desire for more specialist information. KF cases are suitable for presenting a wide range of diseases and for training students' clinical decision-making skills. The Clinical Reasoning Tool can be used for better structuring and visualizing the reasoning process.

## Introduction

The most important day 1 competencies in veterinary neurology were identified by Lin et al. (1). In addition to the neurological examination and neuroanatomical localization, the most important diseases of the central and peripheral nervous system of domestic animals were defined as learning objectives (1).

For students the best way of learning these competences and the Clinical Reasoning approach to work up diseases is the participation in clinics and work with real patients. In addition to the discussion of real patients in small group rounds or face-to-face seminars, computer-based e-learning applications can enable an interactive case study and ensure that all enrolled students have the same access to learn specific competences and diseases. The high acceptance of the CASUS^®^ system, a learning and authoring system for the creation of virtual patients (VP), as well as the need for further case-based, interactive course formats could already be demonstrated for veterinary medicine (2, 3). VP could also be established as a successful concept for teaching applied knowledge in basic subjects of veterinary education (4, 5). Among other common definitions of VP, Ellaway et al. describe them as “an interactive computer simulation of real-life clinical scenarios for the purpose of medical training, education, or assessment. Users may be learners, teachers, or examiners.” (6). Especially as a voluntary learning offering (7), the patient scenarios can be combined well with other events in the form of blended learning concepts. Furthermore, the processing of VP promotes the learners' Clinical Reasoning (CR) skills (8).

In addition to the previously widely tested cases in the Long Case Format (LC), short Key feature cases (KF cases) should be offered as part of elective courses. This type of case not only promotes the transfer of procedural knowledge, but also trains and tests students' clinical decision-making (9). Procedural knowledge describes the knowledge of performing a task and thus stands in the knowledge pyramid between purely descriptive factual knowledge and the performance of the task itself (10). Both competencies should be promoted within the framework of this project. The term Key feature coined by Bordage and Page refers to a critical decision point in the solution of a patient case (11). It has been demonstrated that each disease can be compressed into a few essential KFs, which can then be effectively used to test clinical decision-making ability (9, 12). KF cases have been successfully used in summative electronic examinations at the University of Veterinary Medicine Hannover (TiHo) since 2011 (13). No comparable project has yet been initiated for teaching veterinary neurology. For human medicine, KF cases have already been validated as VP for training clinical decision-making in the context of voluntary examinations (14, 15). Neurological KF cases have been created and validated for human medicine. A very high acceptance for this examination format could be demonstrated (16).

To improve CR skills, Hege et al. developed a new concept mapping tool that can be integrated into VP systems (17). While the tool has already been tested in some projects with regard to improving CR in human medicine (17–19), comparable studies for veterinary medicine are not yet available.

The aim of this prospective study was to determine the usage, learning success, usability and acceptance of the KF cases in comparison to the LC format. Furthermore, the additional feature provided in CASUS^®^, the CR tool, was tested for veterinary medicine.

After evaluation of the new concept by the students, the course format should serve as a validated prototype for further courses at the university.

The following hypotheses should be tested:

1. Neurological KF cases are well accepted, the time required to work on a case is appropriate in comparison to the increase in knowledge (based on subjective self-evaluation) by students.
2. Student's acceptance of the KF format is higher compared to Long Cases.
3. With the KF format, a larger number of neurological clinical cases can be adequately taught.
4. The integration of the CR tool into CASUS^®^ is a useful augmentation for KF cases and well accepted by students.

## Materials and methods

### Case preparation

At the beginning of the project, 38 neurological KF cases were created based on the suggestions that Lin et al. (1) had created for veterinary medicine. The teaching and learning management program CASUS^®^ was used to create the cases. In order to conceptualize the KF cases in a useful way, especially with regard to the CR approach, advice on the correct elaboration of the questions was followed (10, 20, 21). Each KF case consists of 3 case cards with additional start and end pages with general notes. The cases were created in a linear format. The critical decision points were mainly developed in exchange and after expert review with Diplomates of the European College of Veterinary Neurology (AT, HV). The most important points were defined, partly adapted, or modified after review (1, 22, 23). In addition a formal review (ES, CK) was also carried out in comparison with the recommendations from the literature (10, 20, 21).

The most commonly used question types were MCQ questions, matching questions and short free-text tasks. The default settings in the course were such that at least 50% of the tasks had to be answered correctly for a case to be assessed as passed. A case repetition was not planned for the better evaluability of the data.

**Figure 1:**
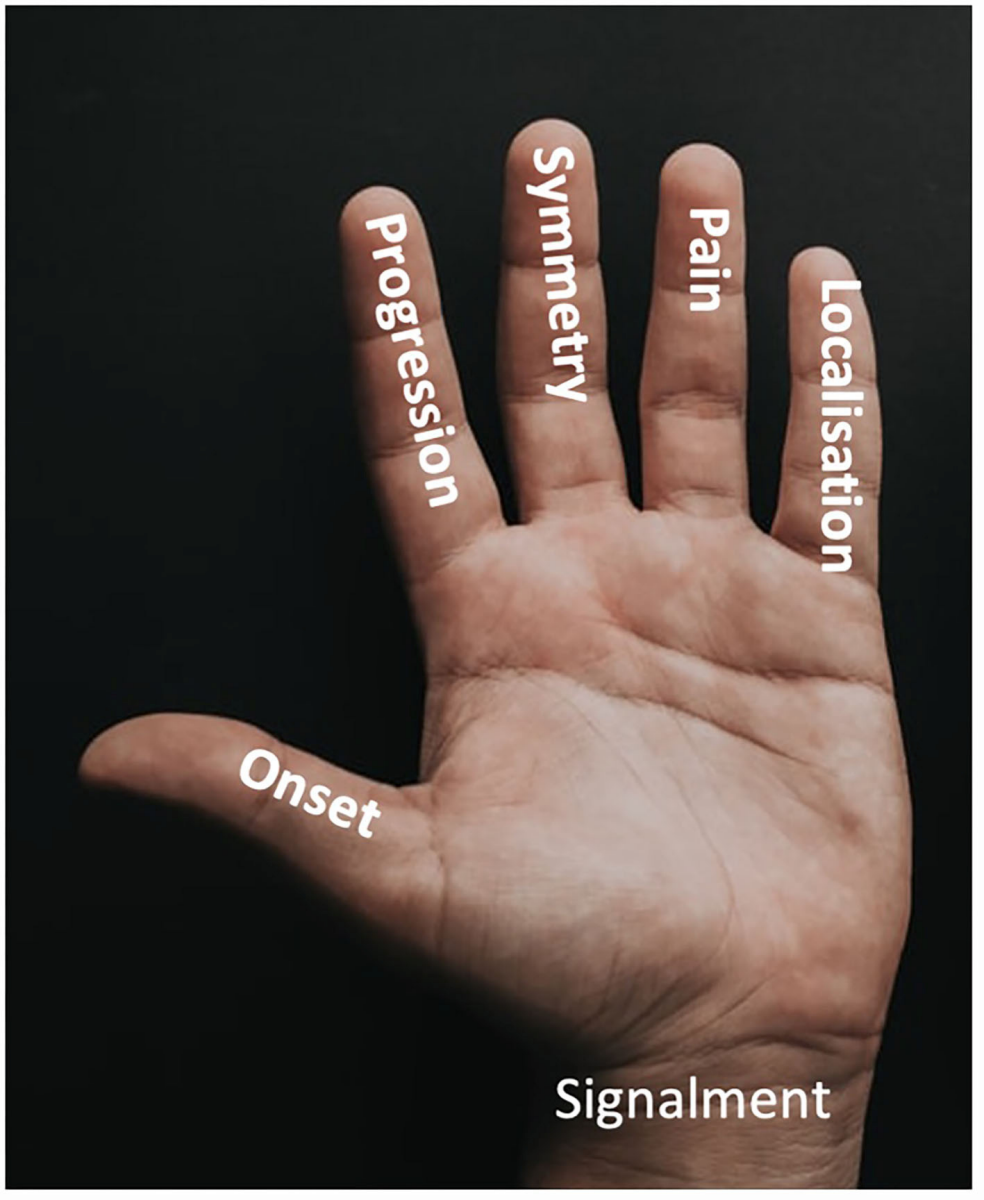
5-finger-rule to characterize a neurological problem and to help to create a list of potential differential diagnoses. According to the Clinical Reasoning approach, information is collected in a problem-oriented manner on signalment, onset, course, symmetry, pain, and neuroanatomical localization, which is then considered in conjunction with the signalment to create a differential diagnosis list.

The first three basic cases deal with the neurological examination, a scheme for reflecting upon differential diagnoses (VITAMIN D scheme) (21) and the 5-finger rule for Clinical Reasoning (24). In several studies evaluating the 5-finger rule (onset, clinical course, symmetry of clinical signs, pain and neurological localization) and signalment for Clinical Reasoning specific patterns were identified to help in diagnosing and differentiating neurological diseases in dogs or cats (see Figure 1) (22, 23, 25–28). Furthermore 35 patient cases on the most frequently occurring diseases of the central and peripheral nervous system as well as myopathies could be created. These diseases are considered to belong to day 1 competences for students of veterinary medicine (1). Real patients treated in the Clinic for Small Animals of the TiHo served as background for the virtual cases. From these patients short video clips, data of clinical examination, laboratory data, data of advanced imaging etc. were taken. Data were used with written owner consent. For each case, a content review was carried out by two board certified neurologists and a didactic and formal review by veterinarians from the Center for E-Learning, Didactics and Educational Research (ZELDA) of the TiHo.

### Clinical Reasoning Tool

Authors of the CASUS^®^ platform developed a special CR tool for integration into VP systems (17). Students can document their clinical decision-making process in a concept map consisting of four fields (“Relevant findings”, “Differential diagnoses”, “Examination/test” and “Therapy”). In addition, a fifth field offers the possibility to enter a summary. Links can be created between individual terms selected from a drop-down menu.

**Figure 2:**
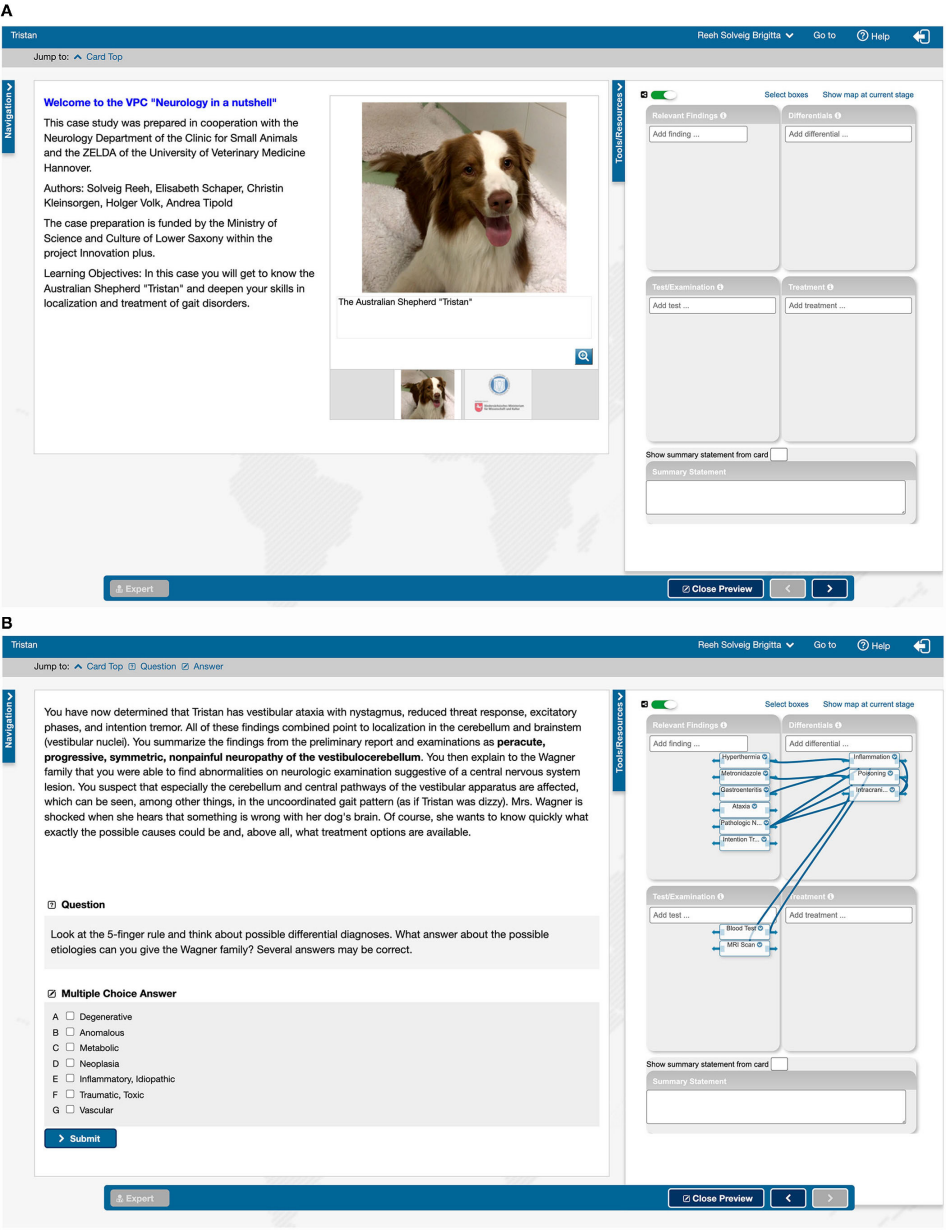
Introductory page **(A)** and penultimate page **(B)** of the CASUS^®^ case “Australian Shepherd Tristan”. **(A)** On the left side an introduction with a picture of the patient; **(B)** on the left side a text sample with background information and a multiple choice task; **(A,B)** on the right side the Clinical Reasoning Tool opened with the fields for findings, test, differential diagnoses and therapy, in **(A)** before processing, in **(B)** filled with terms.

This new tool was used for 8 cases (see Figure 2). These cases were compared to 8 cases without the use of the CR tool characterizing a similar disease/learning objective with comparable tasks.

### Study design

The new elective courses were designed in the following way: Cases were used for two courses in blended learning format. For the first course in the winter term 2019/2020, referred to as VPC (Virtual Patient Course) 2 in the following, 17 cases were provided. The second course (referred to as VPC 3) followed during summer term 2020 with a total of 23 cases presented, including 3 basic cases (neurological examination, VITAMIN D scheme and 5-finger-rule) and 20 patient presentations, of which 8 could be worked on using the CR tool. The different presented diseases are summarized in Table 1. The target group were students of the clinical semesters of the University of Veterinary Medicine Hannover (3rd and 4th year students).

**Table 1:** List of all created key feature cases with the name of the topic field, the disease and the number of cases per category.

**Topic areas**	**Key feature cases**	**Number**
Basic cases	The neurological examination, VITAMIN D scheme, 5-finger rule	3
Cerebrum	Idiopathic epilepsy, structural epilepsy, reactive seizure disorders (portosystemic shunt, hypoparathyroidism, poisoning), hydrocephalus, thiamine deficiency, cortical necrosis	10
Spinal cord	Intervertebral disc herniation (various locations), atlantoaxial subluxation, vertebral luxation, acute non compressive nucleus pulposus extrusion (ANNPE), cervical spondylomyelopathy, degenerative myelopathy, degenerative lumbosacral stenosis (cauda equina syndrome), lymphoma	11
Vestibular diseases	Otitis media/interna, idiopathic/geriatric vestibular syndrome, Metronidazole intoxication	3
Peripheral nerves and muscles	Cushing myopathy, myasthenia gravis, tetanus, masticatory muscle myositis, polyradiculoneuritis	6
Inflammatory	Steroid-responsive meningitis-arteritis (SRMA), meningoencephalitis of unknown origin (MUO), idiopathic cerebellitis, cerebellar hypoplasia after parvovirus infection, feline infectious peritonitis (FIP)	5
Total		38

*N* = 38; *N*, number.

In addition to CASUS^®^ platform, a course room was set up on the free available learning management system Moodle to provide information material (e.g., publications, book chapters, further results and data of patients, videos on the course of the disease). The aim was to provide expert knowledge beyond the course with short KF cases for those students particularly interested. The Moodle entries were linked directly to the cases in CASUS^®^ and could be accessed on a voluntary basis during or after the case work.

At the beginning and end of each course, participants were invited to join meetings to discuss technical and content-related procedures and questions. These meetings were initially face-to-face classes, later performed *via* Microsoft^®^ Teams (Microsoft Corporation, California, USA) due to the COVID-19 pandemic.

During the course, the students were able to contact the lecturers and each other *via* the comment function in CASUS^®^, *via* e-mail or *via* the learning management system Moodle. There was the possibility to discuss content-related questions individually or in the group as well as to give direct feedback on individual case cards.

### Evaluation and statistical analysis

After the end of each course, an evaluation was carried out by means of an online questionnaire in the survey tool LimeSurvey^®^ (LimeSurvey GmbH, Hamburg, Germany). The invitation links were sent to all participants *via* e-mail. The students could participate within a period of 2 weeks. The basis for the survey was a validated questionnaire on Virtual Patients (29) which was supplemented by own questions on the use of the CR tool as well as questions in free text format. The additional questions were validated by co-workers of ZELDA. The respondents could rate statements on course organization, authenticity of virtual cases, learning effect and learning climate on a 6-point Likert scale (1. strongly agree, 2. agree, 3. somewhat agree, 4. somewhat disagree, 5. disagree, 6. strongly disagree). Mean values and standard deviations were calculated from the results of the evaluations for statistical comparison. In addition, all free-text answers were categorized for qualitative and quantitative thematic content analysis.

After course completion, the participants' results were exported from the CASUS^®^ system to Microsoft^®^ Office Excel 2010 (Microsoft Corporation, California, USA) and used for statistical analysis.

To allow comparison of KF cases with cases in LC format, the results of a course with long cases (referred to as VPC 1) that took place in the summer term 2019 were analyzed. In the long cases as well as in the KF cases similar question types (e.g., multiple choice answer, sorting answer, matrix answer) were used. The long cases consisted of 18–21 individual cards on which all information about the case concerning general examination, all special examinations, treatments as well as etiology and pathophysiology of the disease were worked out. On some cards additional information has been made available under the tab “Expert Knowledge” for those particularly interested. In contrast, only selected aspects of the disease were highlighted on the three cards of the KF cases.

This elective course with 9 cases with neurological diseases in LC format was evaluated with the same validated survey questionnaire as the KF courses. For this course, too, the analysis data were exported from the CASUS^®^ system to Microsoft^®^ Office Excel 2010 (Microsoft Corporation, California, USA) and used for statistical analysis.

A descriptive evaluation of all data in Microsoft^®^ Office Excel 2010 (Microsoft Corporation, California, USA) was followed by the investigation of individual questions with the help of the statistical program SAS Enterprise Guide Version 7.15 (SAS Institute Inc. Cary NC, USA).

After testing for normal distribution, pairwise comparisons were made using *t*-test or Wilcoxon test depending on the results. To examine more than two independent samples, the Kruskall-Wallis test was used for non-normally distributed data.

### Ethical statement and data protection

This study was conducted according to the ethical standards of the University of Veterinary Medicine Hannover, Foundation. The entire project was approved by the university's data protection officer. The voluntarily participating students consented to the processing of their data in accordance with the EU General Data Protection Regulation of 2018 (General Data Protection Regulation Art. 6 I 1 lit. e i.V.m. 89 and Lower Saxony Data Protection Act § 3 I 1 No. 1 NHG, § 13). All collected data were evaluated and processed anonymously in accordance with the data protection regulation of the university. Data from patients were used after written owners consent.

## Results

### Key feature cases

A total of 38 KF cases were prepared for the two elective courses. The cases “The neurological examination”, “VITAMIN D scheme” and “5-finger rule” repeat the basics for solving neurological cases in dogs and cats and thus serve as tools for working on the actual VP. The other 35 KF cases deal with diseases of the cerebrum (*n* = 10), myelopathies (*n* = 11), diseases with vestibular signs (*n* = 3), pathologies of the peripheral nervous system or muscles (*n* = 6) and (multifocal) lesions of inflammatory origin (*n* = 5). Some cases could theoretically be listed in more than one category, but for reasons of clarity each case is listed only once (see Table 1). For each case learning objectives were defined.

### Participants

**Figure 3:**
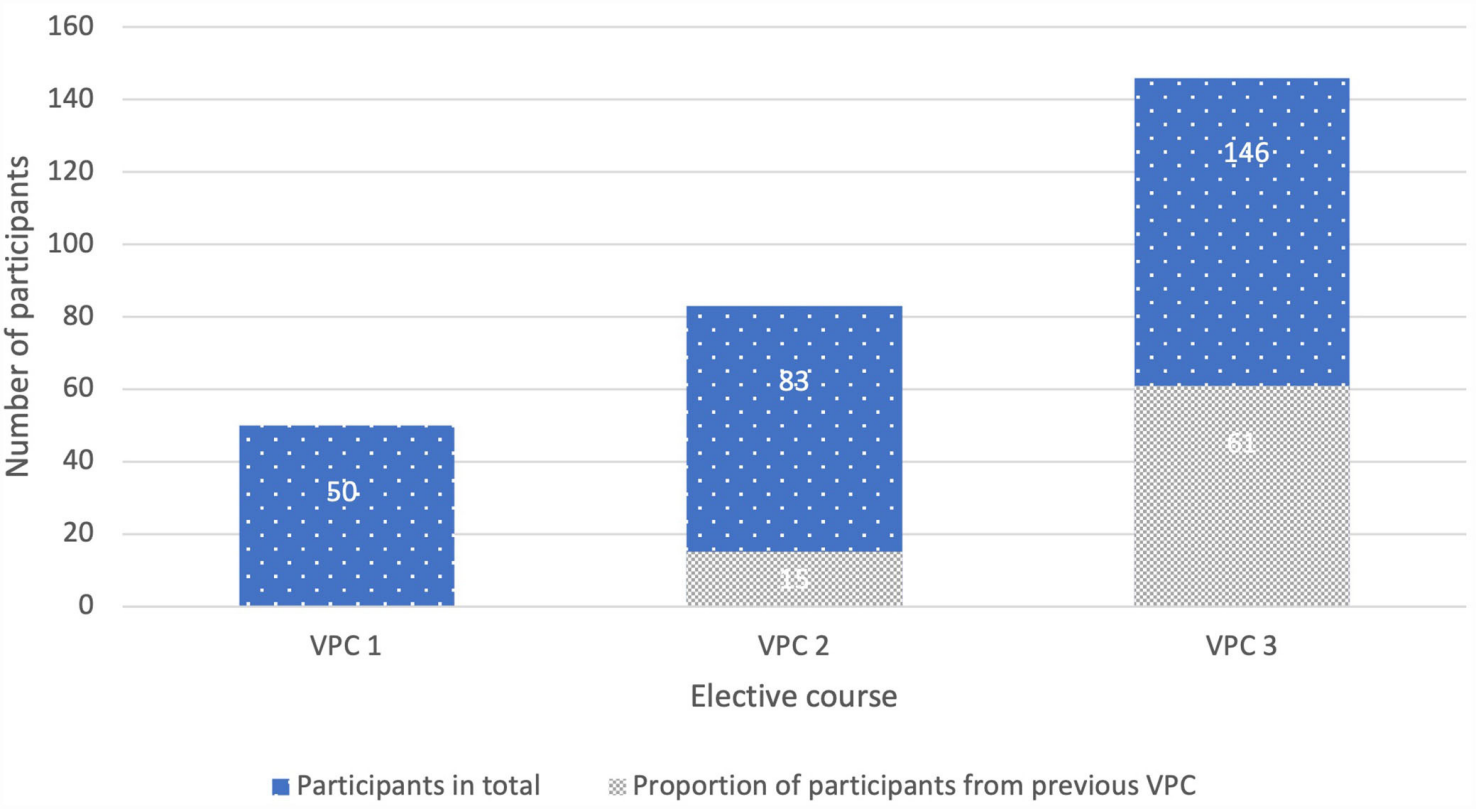
Total participants and proportion of participants taking the previous VPC, VPC 1 (*N* = 50, 20 3rd year and 30 4th year students), VPC 2 (*N* = 83, 39 3rd year and 44 4th year students), VPC 3 (*N* = 146, 74 3rd year and 72 4th year students); VPC, virtual patient course; *N*, number.

Fifty students participated in a CASUS^®^ elective course with 9 neurological cases in the LC format in summer term 2019. These 9 VP have been used for several years and were not created specifically for this project. The results of this course (VPC 1) were used for comparison with the KF cases. For the first KF course “Neurology in a nutshell” in the winter term 2019/2020, VPC 2, 83 students registered. The second KF course in summer term 2020, VPC 3, was chosen by 146 students. In total, the new KF elective course was attended by 229 veterinary students from the 3rd to 4th year (see Figure 3).

The casework of VPC 1, 2 and 3 is shown in Table 2.

**Table 2:** The table shows for each VPC the absolute and relative number of processed cases per student and successfully processed cases per student.

	**VPC 1**	**VPC2**	**VPC 3**
Average number of cases processed per student	8.20 (88.89%)	15.00 (88.24%)	19.38 (84.26%)
Average number of successfully processed cases per student	6.38 (70.89%)	11.14 (65.53%)	14.02 (60.96%)

VPC 1 with 9 LCs (N = 50), VPC 2 with 17 KFs (N = 83), VPC 3 with 23 KFs (N = 146). VPC, virtual patient course; N, number; LC, long case; KF, key feature.

### Comparison of the casework results

**Figure 4:**
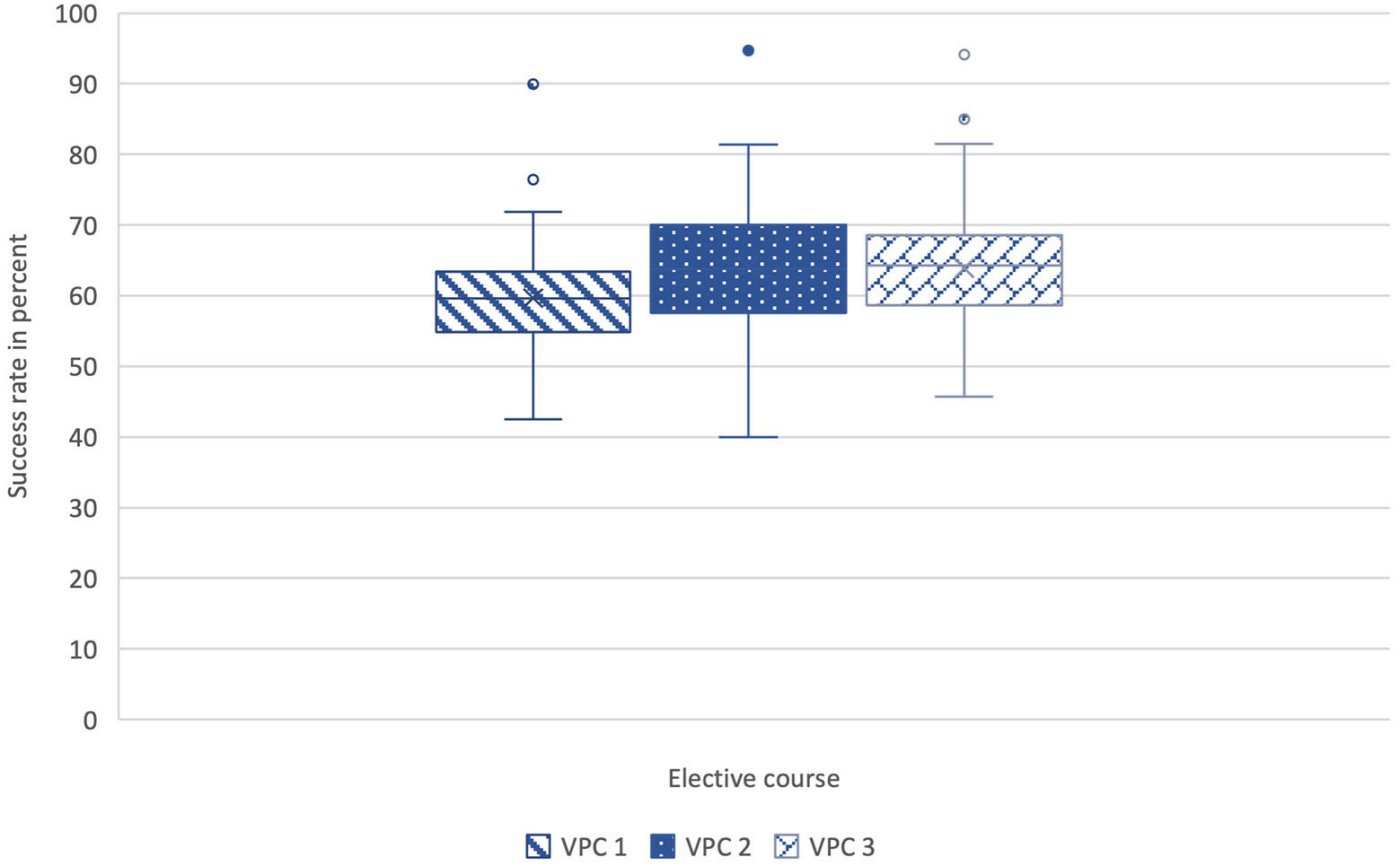
Comparison of the mean values of the success rates (%) of all cases per student between VPC 1 (*N* = 50), VPC 2 (*N* = 82) and VPC 3 (*N* = 146). All box plots indicate minimum score, first quartile, median, mean, third quartile, maximum score, and outliers. The pairwise comparison of means between the success rates of the three VPCs did not reveal any significant differences (*p* > 0.05). VPC, virtual patient course; *N*, number.

To analyze the success rates of participants between the individual courses, the mean values of the total number of caseworks per student were compared. A case was evaluated as successfully completed if more than 50% of the questions were answered correctly. The range of success rates in VPC 1 is between 42 and 90%, the mean value is 60%. The values for VPC 2 are between 40 and 94%, here the mean value is 64%. In VPC 3 the percentages varied between 45 and 94%, the mean value is 64% (see Figure 4). The pairwise comparison of means between the success rates of the three VPCs did not reveal any significant differences (*p* > 0.05).

**Figure 5:**
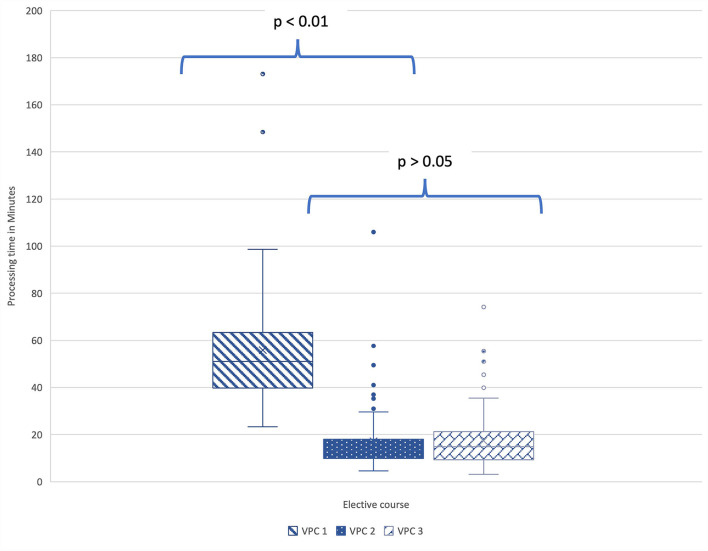
In the pairwise comparison between the values of the processing times (min) of the three VPCs in the Kruskall-Wallis test, there was a significant difference between the processing times of VPC 1 and VPC 2 (*p* < 0.01) as well as VPC 1 and VPC 3 (*p* < 0.01). VPC 1 (*N* = 50), VPC 2 (*N* = 82), VPC 3 (*N* = 146). All box plots indicate minimum score, first quartile, median, mean, third quartile, maximum score, and outliers. *N*, number; VPC, virtual patient course.

The required processing time in minutes per student and case was examined. For this purpose, an average processing time was determined for all cases for each student. These values were finally compared across all VPCs. In VPC 1 working on the long cases, the students needed between a minimum of 23 and a maximum of 99 min for processing. The mean value was 56 min. In the first KF elective, VPC 2, the mean value of the processing time was 17 min. A minimum of 5 and a maximum of 30 min were needed to solve the cases. The situation was similar for VPC 3. On average, students needed 17 min to solve a case. The minimum was 3 and the maximum 35 min (see Figure 5). When comparing the data of the three VPCs in the Kruskall-Wallis test followed by pairwise comparison in the posthoc test, there was a significant difference between the processing times of VPC 1 and VPC 2 (*p* < 0.01) as well as VPC 1 and VPC 3 (*p* < 0.01). The calculated effect size was strong (*r* = 0.89; *r* = 0.65). The processing times of the two KF-VPCs 2 and 3 did not differ significantly from each other (*p* > 0.05).

### Result analysis of students with multiple course attendance

Looking at the success rates of students who attended multiple courses, it is noticeable that students who had previously attended VPC 1 with the LCs did significantly better in the follow-up courses VPC 2 or VPC 3. Participants who had taken VPC 2 were not more successful in VPC 3 (see Table 3). The comparison of the results of students who attended KF courses repeatedly with the results of their fellow students who attended a course for the first time does not show any significant differences. In this analysis, success is given as a percentage of correctly answered questions.

**Table 3:** Overview of students who attended several VPCs with details of the VPC, number of students, mean values of the percentage of correctly answered questions of the respective VPC and *p*-values in the comparison of success rates (success was given as the percentage of correctly answered questions per student in the case cards).

**VPC participation**	**Number of students**	**Mean values of the percentage of correctly answered questions of the respective VPC**	**Comparison of success rates (*p*-Value)**
VPC 1 then VPC 2	15	VPC 1: 58.4% VPC 2: 66.3%	<0.01
VPC 1 then VPC 3	11	VPC 1: 57.8% VPC 3: 66.0%	0.013
VPC 2 then VPC 3	61	VPC 2: 65.1% VPC 3: 63.7%	0.376

VPC, virtual patient course.

### The Clinical Reasoning Tool

For VPC 3, a total of 8 cases were created with the integration of the CR tool. Expert answers were entered for the four fields (findings, diagnoses, examinations/test, therapy) during case creation. The default settings were chosen so that the students did not have access to the expert entries during case processing but could compare them with their own answers once the case was completed. The 8 cases with the CR tool were contrasted with 8 similar cases without using the tool for the evaluation. Compared cases included diseases from the same category or with similar clinical signs and had the same or similar differential diagnoses. Therefore, similar questions could be asked on each card for comparability (see Table 4).

**Table 4:** Comparison of cases with and without integration of the Clinical Reasoning (CR) Tool in VPC 3.

**Case created without CR-Tool**		**Case created with CR-Tool**	
**Case number**	**Disease**	**Case number**	**Disease**
1	Degenerative myelopathy	1	Degenerative lumbosacral stenosis (cauda equina)
2	Portosystemic shunt	2	Hypoparathyroidism
3	Rhodenticide intoxication	3	Metronidazoline intoxication
4	Feline infectious peritonitis	4	Lymphoma
5	Steroid-responsive meningitis-arteritis (SRMA)	5	Atlantoaxial subluxation
6	Meningoencephalitis of unknown origin (MUO)	6	Intervertebral disc protrusion (C1–C5)
7	Vertebral luxation	7	Intervertebral disc herniation
8	Structural epilepsy	8	Idiopathic epilepsy

VPC, virtual patient course.

After completion of VPC 3, the results of the compared case pairs were examined with regard to the processing time required and the success rate achieved. A case was evaluated as successfully completed if more than 50% of the questions were answered correctly. Cases without the CR tool were completed 957 times, comparable patient cases with the tool 956 times. In the group without the CR tool, a success rate of 60.3% on average was recorded, while in the group using the tool it was 58.3%. Regarding the success rate, the Wilcoxon test showed that there was no significant difference (p = 0.128) between the results of the two groups.

The processing time of the patient cases without the new application was 15 min on average, while solving with the use of the concept mapping tool took 19 min on average. The Wilcoxon test for two connected samples showed that the processing time for cases with the CR tool was significantly higher than for cases without the tool (*p* < 0.01).

**Figure 6:**
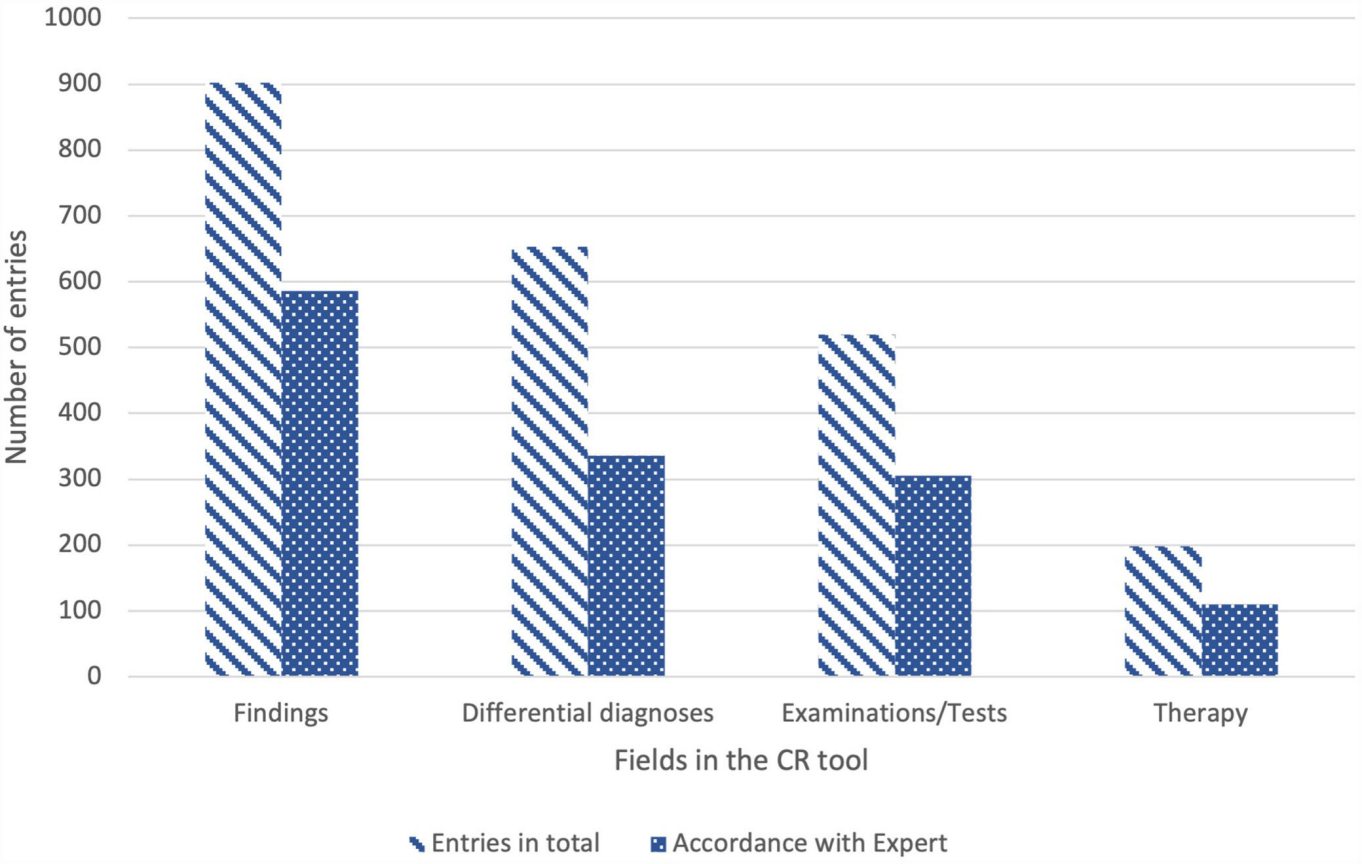
Numbers of entries [students (*N* = 146) could choose as many terms per field as they wanted] in the Clinical Reasoning Tool for the categories findings, diagnoses, examinations/tests and therapy with indication of total entries and entries that correspond to expert statements; *N*, number; CR, Clinical Reasoning.

In addition to processing time and success, data on the use of the tool itself was also evaluated. Between 96/146 (CR case 6) and 130/146 (CR case 2) students used the tool per case. In addition, the entries in all fields of the concept map were examined for each case and the entries were compared with the expert information. Students were able to make as many entries per field as they wanted. They entered 902 findings, of which 65% (*n* = 586) agreed with the expert findings. Differential diagnoses were the second most frequently noted feature in the concept map (*n* = 653), of which 52% (*n* = 336) matched those of the lecturers. For examinations and tests, the course participants selected 520 terms, of which 59% (*n* = 305) were similar to those of the preset expert statements. The fewest entries were made for therapy options (*n* = 198) of which 56% (*n* = 110) corresponded to the expert information (see Figure 6).

### Survey results

The questionnaires for the three VPCs were completed by a total of 246 students. The response rate of survey in VPC 1 was 87% (*n* = 47), in VPC 2 82% (*n* = 68) and in VPC 3 90% (*n* = 131).

### General survey results

The results of all statements on the categories coordination, authenticity, learning effect and learning climate are listed in the in Table 5.

**Table 5:** Means and standard deviations of survey responses on coordination, authenticity, learning effect and learning climate of the course on the 6-point Likert scale (1 = disagree at all, 6 = agree completely), VPC 3 (*n* = 131).

	**Average (standard deviation)**		
	**VPC 1**	**VPC 2**	**VPC 3**
The technical requirement for processing the virtual patients was adequate.	5.2 (0.9)	5.5 (0.5)	5.1 (0.9)
The contents of the virtual patients and those of the corresponding courses complemented each other in a meaningful way.	4.7 (1.1)	5.6 (0.6)	6.7 (0.5)
I had easy access to the Virtual Patients whenever I wanted.	5.6 (0.7)	5.6 (0.6)	5.7 (0.6)
The case was adapted to my level of knowledge in terms of difficulty.	4.0 (0.9)	4.6 (1.0)	5.0 (0.8)
While I was working on the case. I had the feeling that I had to make the same decisions as a vet in real life.	4.7 (0.9)	4.8 (0.9)	4.7 (0.9)
After completing the case. I felt better prepared to treat patients with this condition in real life.	5.0 (1.0)	4.7 (0.8)	4.6 (0.6)
The expert opinion I received was helpful in improving my diagnostic conclusion.	4.7 (1.0)	5.2 (0.8)	5.2 (0.8)
The questions I was asked while working on the case were helpful to improve my differential diagnostic thinking on the case.	4.9 (1.0)	5.0 (0.7)	5.0 (0.7)
After working on this case. I felt better prepared to secure a diagnosis in a real patient and to rule out important differential diagnoses.	4.7 (1.0)	4.6 (0.9)	4.9 (0.8)
The combination of virtual patients and corresponding courses improved my clinical-diagnostic thinking.	4.9 (1.0)	4.9 (0.9)	4.9 (0.9)
After case processing. the most important treatment steps were always clear to me.	4.7 (0.9)	4.4 (0.9)	4.8 (0.9)
I would like to see more accompanying information in the cases.	4.2 (1.3)	4.4 (1.4)	3.8 (1.2)
Overall. I experienced an increase in knowledge by working through the cases.	5.5 (0.7)	5.4 (0.7)	5.5 (0.6)
I felt that the learning environment during the course was positive.	5.4 (0.8)	5.5 (0.7)	5.6 (0.7)
I felt part of a group while working on the cases.	2.7 (1.4)	2.3 (1.2)	2.3 (1.1)
I like to work on learning content virtually.	5.0 (0.9)	5.0 (0.9)	4.8 (1.0)
I found the processing time of a case to be reasonable.	4.9 (1.0)	5.4 (0.7)	5.4 (0.7)
I would like to see more learning cases that are shorter and less complex.	3.7 (1.1)	3.1 (1.4)	2.7 (1.3)
I would like more case-based learning.	5.2 (1.0)	5.4 (0.8)	5.4 (0.8)

93.63% (*n* = 44) of the participants from VPC 1 agreed with the statement “The time required for case processing is appropriate in relation to the increase in knowledge”. 6.38% (*n* = 3) of the respondents disagreed with this statement and stated that the processing time was too long in relation to the increase in knowledge. In VPC 2, 98.53% (*n* = 67) of the survey participants agreed with the statement, while 1.47% (*n* = 1) thought the processing time was too long. In VPC 3, most students (99.24% (*n* = 130) also agreed with the statement. One person (0.76%) stated that the processing time was too long in proportion. The difference in the answers to this statement is not statistically significant (*p* > 0.05).

Students who had attended both VPC 1 with the long cases and one of the KF-VPCs were asked to give comparative ranking marks for the courses (range 1 to 6, 1 = very good, 6 = poor). For VPC 1, the average grade was 2.36. VPC 2 received a grade of 1.86, and the last course was given an average grade of 1.15.

### Survey results on Moodle

For the KF-VPCs, the learning management platform Moodle was evaluated based on 8 statements. The statement “The technical access in Moodle was unproblematic” was evaluated in VPC 2 with an average value of 4.8 on the 6-point Likert scale. In the second KF VPC, VPC 3, this value was 5.3. There was also high agreement with the question about the practical relevance of the documents (mean = 5.0). In the statements on the desire for more interaction, no clear approving or disapproving tendency could be found. Further results are listed in Table 6.

**Table 6:** Means and standard deviations of survey responses on Moodle and the Clinical Reasoning Tool on the 6-point Likert scale (1 = disagree completely, 6 = agree completely), VPC 3 (*N* = 131).

**Statements about Moodle**	**Average (standard deviation)**	
	**VPC 2**	**VPC 3**
I would like to see more learning cases that are longer and more complex.	3.0 (1.4)	3.1 (1.2)
The technical access in Moodle was unproblematic.	4.8 (1.2)	5.3 (0.8)
The supplementary course room in Moodle is superfluous.	3.3 (1.1)	3.6 (1.3)
The documents in Moodle for the individual cases were helpful for my understanding.	4.2 (1.0)	4.2 (1.0)
The documents had a practical relevance.	5.0 (0.8)	5.0 (0.9)
The course room in Moodle is a useful supplement to the CASUS^®^ case studies.	4.1 (1.2)	4.0 (1.2)
I would like to see more interaction between the participating students.	2.8 (1.3)	2.7 (1.2)
I would like to see more interaction between the participating students and the lecturers.	3.0 (1.3)	3.0 (1.2)
**Statements about the CR Tool**	**Average (standard deviation)**	
	**VPC3**	
I always used the CR tool in the cases where it was available.	3.0 (1.6)	
The technical operation of the CR-Tool worked without any problems.	4.2 (1.3)	
I would like to see more of the short key feature cases with CR tool limited to three key questions.	3.2 (1.2)	
I would like to see more of the short key feature cases limited to three key questions without CR tool.	4.1 (1.3)	
The CR tool was helpful to better structure the information about the patient.	3.3 (2.0)	
I felt that I could solve the cases better by using the CR tool.	2.8 (1.1)	
The CR tool is superfluous.	4.0 (1.3)	

N, number; VPC, virtual patient course.

### Survey results on the Clinical Reasoning Tool

In the third survey, statements on the newly implemented CR tool were also evaluated for the first time. With mean values between 2.8 and 4.2, most of the statements were answered rather neutrally. A relatively high score (mean = 4.2) was achieved for the statement on the ease of use of the tool. Most students disagreed (45%) that they were able to solve the cases better by using the CR tool (mean = 2.8). Further results are shown in Table 6.

### Free text answers

**Figure 7:**
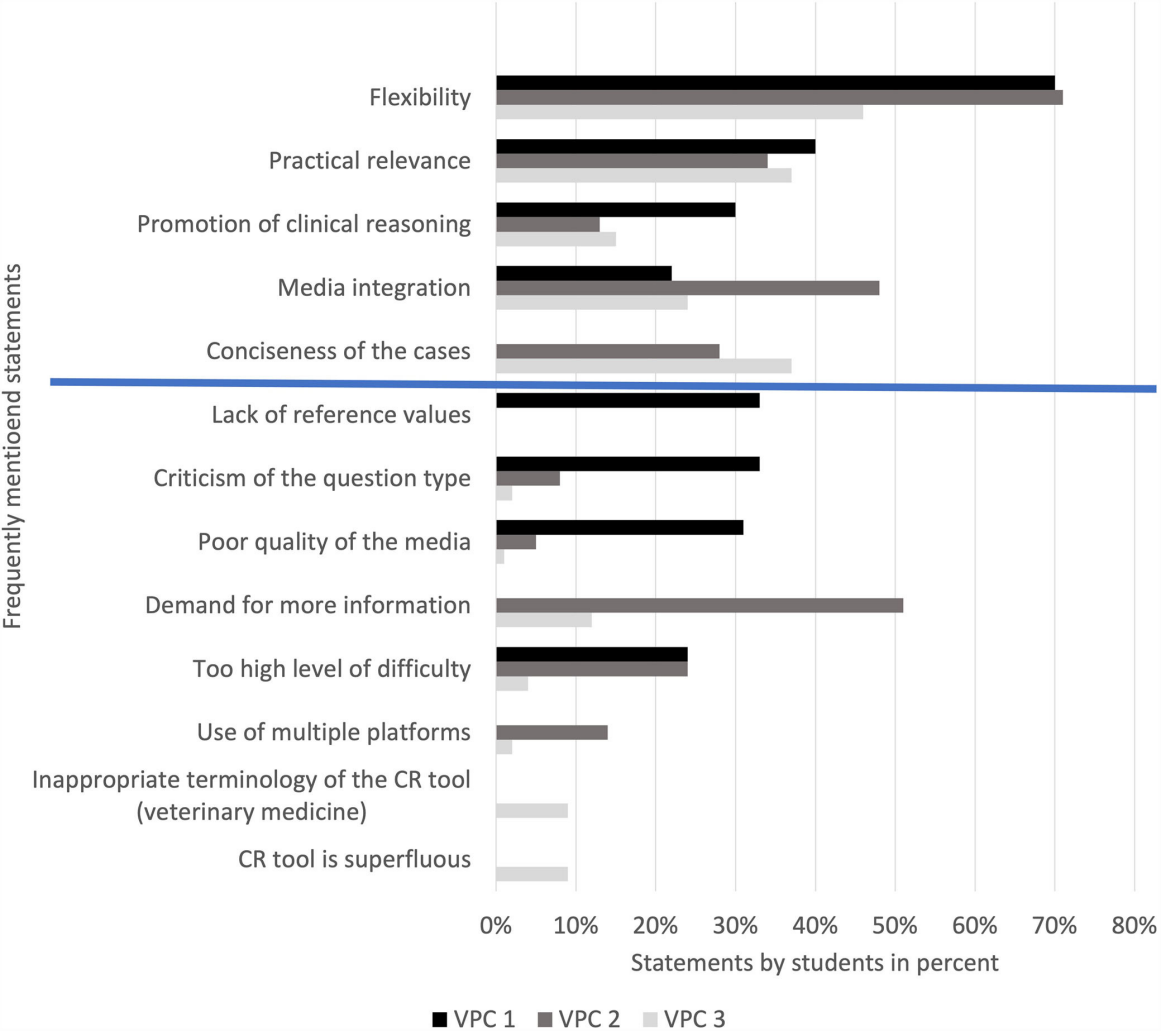
Proportion of students per survey providing positive and negative feedback, VPC 1 (*N* = 45), VPC 2 (*N* = 37), VPC 3 (*N* = 127). The first five items include positive feedback (above blue line), the last eight negative feedback (below blue line). VPC, virtual patient course; *N*, number.

At the end of each survey, two open questions were asked about positive and negative feedback. For descriptive evaluation, all statements made were categorized according to content. In the positive feedback the flexibility of the course format and the practical relevance of the cases was emphasized in each survey. Other frequently mentioned categories are listed in Figure 7.

The negative feedback differed more clearly between the three course formats. After VPC 1 the lack of reference values in the tasks, the open questions and the quality of the media were criticized above all. For the KF-VPCs, the respondents wished for more information and expert knowledge. In VPC 3, many students also criticized the CR tool, mainly regarding the given terminology of the dropdown menu. Further categories of negative feedback are shown in Figure 7.

## Discussion

The aim of this project was to test Key feature cases as virtual patients with neurological diseases using a new Clinical Reasoning Tool with regard to learning success, usage, usability and acceptance for veterinary teaching.

The demands on veterinary medical education have undergone a significant change in recent years, which has been expressed by the regular adaptation of the German Ordinance concerning the Certification of Veterinary Surgeons (TAppV) (30). In addition to linking theoretical and clinical knowledge, paragraph 2 of the ordinance also calls for the use of interactive learning programs (31). The most important “first-day competences” have been defined by various supervisory bodies and commissions (32–34) and at the same time the need for alternative, practice-oriented teaching concepts has been anchored in the curricula. The classic courses (lecture, seminars or practical classes) are increasingly supplemented by innovative offerings, such as skills labs for training practical skills (35). These offerings also include digital formats using e.g., virtual patients (36, 37).

The present project represents an expansion of the offering of VP at the TiHo, in which KF cases and the CR tool were used for the first time as part of elective courses.

### Participants

The acceptance of the digital teaching format by the students was very high. The number of participants increased significantly from 83 students in VPC 2 to 146 students in VPC 3. Of all students in the clinical years (*N* = 523) a total of 28% (*n* = 146) participated in this special educational offering. From the 83 participants in VPC 2, 73% (*n* = 61) decided to also attend the follow-up course VPC 3. In VPC 1, 40% (*n* = 20) third-year and 60% (*n* = 30) fourth-year students participated; in VPC 2, 47% (*n* = 39) third-year students and 53% (*n* = 44) fourth-year students participated; in VPC 3 it was 51% (*n* = 74) third-year vs. 49% (*n* = 72) fourth-year students. It can be summarized that the distribution of students from different years is comparable, in VPC 1 proportionally slightly more students from higher semesters participated.

This high participation rate is a very positive result, especially with regard to the frequently discussed “neurophobia” of students (38). Focusing the neurological curriculum on essential content has been described as a measure to prevent neurophobia (39). By integrating a previously created veterinary curriculum (1) into the case planning as well as by the concise structure of the cases, a focus on the most important contents (KFs) could be achieved, which in turn possibly increased the motivation for the subject and facilitated learning.

### Success rate and processing time

The total number of successful case processing as well as average case processing per person were slightly higher in VPC 1 than in the KF-VPCs (see Table 2). Even though the differences are only minor, one could assume that the Long Cases courses were mainly chosen by students who have a special interest in neurology and like to learn about the diseases in detail. Meanwhile, the title of the other VPCs “Neurology in a nutshell” possibly appealed to a broader spectrum of students, which is reflected not only in the overall higher numbers of participants, but also in the slightly lower results. Considering the success rates of the individual students, the greater dispersion of the results in KF-VPCs supports this assumption. Different degrees of tasks difficulties could be another reason to explain this variance. On the other hand, the mean values of the success rates for the KF-VPCs are slightly higher (64%) than for VPC 1 with the long cases (60%). Similar results have already been obtained in previous studies. The success rates between 58.8 and 75% determined by Hatala and Norman (40) for examinations in the key feature format correspond to the observations of the present study, in which average success rates of 64% were found for the KF-VPCs.

In a study with longer CASUS^®^ cases (average processing time 43 min) by Simonsohn and Fischer, the average success rate was 44% (41). Even though the comparability of the studies is limited due to the different study design, this observation tends to coincide with the results from VPC 1, in which the success rate of 60% on average is lower than that of the KF-VPCs.

As expected, solving the longer cases took considerably more time on average (56 min) than solving the KF cases (17 min). Outliers upwards in the processing times for the short cases can be plausibly explained by various approaches: On the one hand, students may have been busy with other activities while simply leaving the case open. On the other hand, it is possible that the particularly interested students studied all the additional material provided for the cases intensively and needed considerably more time for this than for the pure case solution. In a study by Hatala and Norman on KFs, the students solved 15 cases with 1–4 cards within 3 h (40), whereas in our study 17 min were needed per case. In another comparative study of different VP systems, the average time needed for learning cases in CASUS^®^ was 20 min. In other learning systems, the time varied between 15 and 45 min, but the length of the individual cases is not discussed in detail (42). All in all, the processing time of short learning cases determined by the current study is within the range of already described results of previous studies.

The data from success rates and processing times suggest that KF cases are solved in less time than LCs with similar success. It can therefore be assumed that a wider range of neurological disorders can be covered by KF cases than in sessions with very detailed cases. Schuwirth and van der Vleuten (43) precisely emphasize this effect in relation to electronic examinations, by pointing out that a large number of cases can be examined per hour through KFs.

Evaluating those participants who first attended VPC 1 and later VPC 2 or VPC 3, it is noticeable that they were able to achieve significantly better results in the follow-up courses, while no significant difference can be found in the intersection of students who only attended the KF-VPCs. One can assume that students who have dealt with cases in detail can later apply their acquired knowledge well in a problem-oriented manner in the shorter cases. In order to measure a real learning effect, however, a study design with pre- and post-tests and several cohorts would have to be chosen, which has not yet been done in this project.

### The Clinical Reasoning Tool

The term Clinical Reasoning is defined differently in various contexts (44, 45). Norman describes CR as “processes doctors use to arrive at an initial diagnosis based on history and physical examination (and occasionally investigations)” (46). Although clinical decision-making training has gained importance in the last years, there is still a need for further integration of relevant concepts (e.g., VP) into curricula in human medicine teaching (47). Especially regarding VP, the same can be assumed for veterinary medical education (48).

Maddison et al. (24) introduce the concept of inductive Clinical Reasoning as a framework for solving veterinary case presentations. This involves creating a problem list where the problems should be defined, prioritized, and specified as precisely as possible. The next steps are to identify the organ system involved, find the exact location, and finally determine the lesion itself using the right questions and appropriate diagnostic tests. With the help of simple questions, beginners in particular can better structure and apply their knowledge and quickly and effectively set up differential diagnoses despite little experience. This concept differs significantly from pattern recognition, which is based on long professional experience and may be prone to errors (24).

Hege et al. developed a corresponding tool to improve the clinical decision-making process of students for implementation in VP systems. In initial studies on the use of the CR tool, in which individual concept maps were analyzed, it was found that students did use the tool, but made fewer entries than the experts (17). In this project, the concept maps created were not examined in detail, but data on the general use of the tool was evaluated. It was noticeable that overall, a large proportion of the students (96–130 of 146) used the tool. Most entries were made on clinical findings (*N* = 902), while only a few terms were selected in the field on therapy and management (*N* = 198). These results are more or less in line with those of Hege et al. who also found that the fewest entries were made in the field of therapy. Differential diagnoses were entered most frequently, followed by findings and examinations (17). It is possible that students in the 3rd and 4th year find it easier to firstly note what they see, while the use of medication and other therapy options cannot yet be recalled in an application-oriented manner. The way the cases were conceptualized in the presented elective courses, in which emphasis was placed on correct findings based on image and video material, may also contribute to this result. The same exercise with students of the practical year (5th year) could be investigated to learn about the influence of the study year on the use of the CR tool. The accordance of the entries with those of the experts was between 52 and 65% for all fields. The true value can be assumed to be slightly higher here, since synonyms, i.e. answers that are correct in meaning, were not included in this calculation.

When comparing the cases with and without the CR tool in terms of success rates it was noticeable that the success rates were slightly higher in the cases without the use of the tool (58.3% with the CR tool vs. 60.3% without the CR tool; *p* > 0.05). A case was considered successfully completed in this analysis if more than 50% of the questions were answered correctly. Comparing the processing time, on the other hand, revealed, that the time needed was significantly higher in the cases with the tool (19 min with the CR tool vs. 15 min without the CR tool; *p* < 0.05). Hege et al. (19) found a similar processing time (22.8 min) for VP with the CR tool. Thus, no direct effect of the tool on the success of case processing could be found in this first experiment. It should be noted, however, that although the authors felt that the contrasted pairs of cases dealt with similar diseases and similar clinical findings, a certain subjective influence on the study is feasible. In a further study, the same 8 cases could be presented in two groups, one using the CR tool and the other not.

In the survey, the statements about the CR tool were rated with values in the middle, rather uncertain range of the 6-point Likert scale. The students tended to agree (mean = 4.2) that the technical operation had worked without problems. In contrast, they tended to disagree (mean = 2.8) that they were able to solve the cases better by using the tool. This result is also comparable with the responses to statements on the usefulness of the tool on a 6-point scale in the evaluation by Hege et al. (17), in which the mean values ranged between 2.8 and 3.2. Some critical comments were also given in the free text responses on negative feedback. While some found the tool fundamentally superfluous (*n* = 12), others criticized that the terminology of the drop-down menu is too much influenced by human medicine (*n* = 12), which led to frustration.

There were some positive comments on the basic conceptualization of the tool. For example, one student commented: “I have processed all cases with the CR tool and also used it. In general, I think it's a great idea for working on cases, because you have all the important information at a glance. I now also use this method to work up cases for report writing or learning.” This statement illustrates that the CR tool can help to provide a framework for writing information on diseases and can also be used beyond a course as a thought-provoking tool by students. Illness scripts, as a process of CR, are mental summaries composed of different attributes and their interconnections that grow into a specific protocol for individual diseases with each new patient case. In addition to licensed physicians developing scripts automatically, this concept needs to be more firmly embedded in teaching (49).

In summary, the analysis data of the CR tool show moderate to high use, depending on the category of the concept map. The results of the survey imply that the technical usability of the tool works well, but that an extension of the given terms to include veterinary terminology would be necessary in the next step to make the tool even more usable. In addition, the conceptualization of the cases themselves needs to be optimized, as the case design can influence the CR process of students (50).

### Survey

The surveys consisted of several sections. In the first 4 sections, statements on the coordination of the course, the authenticity of the cases and the learning effect and learning climate were rated on the 6-point Likert scale. Overall, mostly high agreement values (mean 4.0–5.5) were given to all statements, whereby no difference between the individual VPCs was discernible for most statements. These results are comparable to those of Grumer et al. who found a high acceptance of neurological KF cases in human medicine using a questionnaire similar to the one used in the present study (16). For veterinary medicine, a good acceptance of KFs in formative examinations has already been demonstrated (13). This result can now also be transferred to teaching formats. The technical operability of the programs functioned without problems, the cases were perceived as close to reality, the learning climate was assessed as positive, and there was also great agreement about the good learning effect of all courses (mean = 5.5; 5.4; 5.5). Overall, almost all students stated that they would like to have more case-based learning (mean = 5.2; 5.4; 5.4). It is interesting that in the KF-VPCs, more affirmative answers (mean = 5.4; 5.4) were given to the statement “I found the processing time of a case to be appropriate” than in VPC 1 (mean = 4.9). This statement also corresponds with the answers to the question of whether the time spent was appropriate in relation to the increase in knowledge. In VPC 1 93.63% of the respondents agreed, in VPC 2 98.53% consented and in VPC 3 it was 99.24% of students that agreed. The difference in the answers to this statement is not statistically significant (*p* > 0.05). A comparison of the school marks given by students who attended several VPCs shows a preference for the KF VPCs (VPC 2 = 1.86; VPC 3 = 1.15) over the VPC with long cases (VPC 1 = 2.36). However, it should be noted that some of the points from the negative feedback on VPC 1 have already been implemented in the KF courses and this may have resulted in less negative feedback on the shorter cases. Despite this difference, VPC 1 also received very good ratings in the other statements, which shows that both VP formats are well accepted by students.

The evaluation and categorization of the free-text answers also provided interesting insights. While the statements of the positive feedback were largely similar between the VPCs, there were higher differences in the negative feedback.

In the first place in the positive feedback, most students in all surveys mentioned the flexibility of the format of all 3 VPCs. This included both the spatial and temporal independence in case processing. The free allocation of time enabled students to optimally integrate the course content into the rest of their schedule. Especially for students with children, this flexibility through digital learning opportunities can be of enormous importance (51). Students also emphasized in all surveys the practical relevance and good preparation for practical activities for later professional life. Explicitly, the promotion of clinical thinking was praised more often in VPC 1 than in the other VPCs, which is surprising, since VPC 3 in particular was designed with this goal in mind. The integration of media was also praised very often, which once again emphasizes the importance of visual material to illustrate VP. The assessment of findings using videos of real patients was intended to make the scenario as real as possible. Some students even expressed that they really felt they had helped the patient, especially when they watched videos of the course of the disease and treatment. Finally, conciseness, i.e., the brevity of the cases, was also frequently mentioned in the KF VPC surveys, confirming the good acceptance of short cases. Some students suggested course formats with a mixture of short and long cases.

In the negative feedback, lack of reference values on laboratory tasks, the quality of the media and the questions (too many open free-text questions) were criticized in particular in VPC 1. In response to this evaluation, special emphasis was placed on improving these three points in the KF VPCs. The successful implementation of the feedback was confirmed by the fact that these categories were mentioned only marginally or not at all in the surveys for VPC 2 and 3. In KF electives, the wish for more information and expert knowledge was expressed in the first place. Albeit the actual intention of short cases is a bundled treatment of the topic, sufficient additional material should be made available at the appropriate place for students especially interested in neurology. Although this was done in the form of surgery reports, examination findings, image and video material or publications on the Moodle learning platform, there seems to be a need for expert knowledge beyond this. A constructive idea of the students was, for example, to create a special, veterinary, neurological glossary.

The excessive degree of difficulty of the cases, which was often noted especially in VPC 1 and VPC 2 with regard to the evaluation of cross-sectional images, illustrates that lecturers sometimes make misjudgments about the previous knowledge of students. After VPC 2, the use of two different platforms, i.e. explicitly CASUS^®^ and Moodle, was criticized. Students found it inconvenient to have to switch between the two systems to access additional information. In VPC 3, fewer statements of this kind were made. This could indicate an increased methodological competence in dealing with Moodle, especially due to the more intensive use in the digital summer term 2020, which was confirmed by higher approval ratings about technical usability (mean = 5.3), than in the previous term (mean = 4.8).

## Conclusion

Overall, with the elective courses on neurological KF cases, a learning offering was created, which was very well accepted by students and could now also be implemented sustainably as an evaluated course offering. An expansion of this validated concept to other disciplines is quite conceivable. The older Long Cases were also well evaluated, produced better improvements in learning in subsequent courses, and seem to continue to be of importance, especially for those explicitly interested in neurology. Courses with a combination of both VP formats would be desirable to motivate the broadest possible mass of students for the subject. The first test of the new CR tool for veterinary medicine functioned technically without major difficulties, but some adjustments for optimal use in veterinary teaching would be necessary for greater acceptance. Using the tool increased processing time, while success rates decreased slightly.

## Data availability statement

The original contributions presented in the study are included in the article/supplementary material, further inquiries can be directed to the corresponding author/s.

## Ethics statement

The studies involving human participants were reviewed and approved by Data Protection Officer of the University of Veterinary Medicine Hannover, Foundation. The patients/participants provided their written informed consent to participate in this study.

## Author contributions

SR, ES, CK, and AT conceived the study and supplemented the already existing questionnaire for the survey. ES, CK, AT, and HV supervised the study. SR created the key feature cases. ES and CK performed the content and didactic review. AT and HV performed the neurological expert review of the cases. SR collected, analyzed, and interpreted the data, which was supervised by ES and CK. SR wrote the manuscript with critical input and notable revisions from ES, CK, AT, and HV. All authors reviewed and approved the final version.

## Funding

This study was supported by the project Innovation Plus of the Ministry of Science and Culture of Lower Saxony.

## Conflict of interest

The authors declare that the research was conducted in the absence of any commercial or financial relationships that could be construed as a potential conflict of interest.

## Publisher's note

All claims expressed in this article are solely those of the authors and do not necessarily represent those of their affiliated organizations, or those of the publisher, the editors and the reviewers. Any product that may be evaluated in this article, or claim that may be made by its manufacturer, is not guaranteed or endorsed by the publisher.
